# Blood-perfused Vessels-on-Chips stimulated with patient plasma recapitulate endothelial activation and microthrombosis in COVID-19†

**DOI:** 10.1039/d4lc00848k

**Published:** 2025-02-25

**Authors:** Huub J. Weener, Thijs F. van Haaps, Ruben W. J. van Helden, Hugo J. Albers, Rozemarijn Haverkate, Heleen H. T. Middelkamp, Milan L. Ridderikhof, Thijs E. van Mens, Albert van den Berg, Christine L. Mummery, Valeria V. Orlova, Saskia Middeldorp, Nick van Es, Andries D. van der Meer

**Affiliations:** a Department of Bioengineering Technologies, University of Twente Enschede The Netherlands H.J.Weener@utwente.nl; b Department of Vascular Medicine, Amsterdam University Medical Center location University of Amsterdam Amsterdam The Netherlands; c Amsterdam Cardiovascular Sciences, Pulmonary Hypertension & Thrombosis Amsterdam The Netherlands; d Department of Anatomy and Embryology, Leiden University Medical Center Leiden The Netherlands; e BIOS Lab-on-a-Chip Group, University of Twente Enschede The Netherlands; f Department of Emergency Medicine, Amsterdam University Medical Center Location AMC Amsterdam The Netherlands; g Department of Medicine—Thrombosis and Hemostasis, Leiden University Medical Center Leiden The Netherlands; h Department of Internal Medicine, Radboud university medical center Nijmegen The Netherlands

## Abstract

A subset of coronavirus disease 2019 (COVID-19) patients develops severe symptoms, characterized by acute lung injury, endothelial dysfunction and microthrombosis. Viral infection and immune cell activation contribute to this phenotype. It is known that systemic inflammation, evidenced by circulating inflammatory factors in patient plasma, is also likely to be involved in the pathophysiology of severe COVID-19. Here, we evaluate whether systemic inflammatory factors can induce endothelial dysfunction and subsequent thromboinflammation. We use a microfluidic Vessel-on-Chip model lined by human induced pluripotent stem cell-derived endothelial cells (hiPSC-ECs), stimulate it with plasma from hospitalized COVID-19 patients and perfuse it with human whole blood. COVID-19 plasma exhibited elevated levels of inflammatory cytokines compared to plasma from healthy controls. Incubation of hiPSC-ECs with COVID-19 plasma showed an activated endothelial phenotype, characterized by upregulation of inflammatory markers and transcriptomic patterns of host defense against viral infection. Treatment with COVID-19 plasma induced increased platelet aggregation in the Vessel-on-Chip, which was associated partially with formation of neutrophil extracellular traps (NETosis). Our study demonstrates that factors in the plasma play a causative role in thromboinflammation in the context of COVID-19. The presented Vessel-on-Chip can enable future studies on diagnosis, prevention and treatment of severe COVID-19.

## Abstract 2

COVID-19 plasma induces endothelial activation and thromboinflammation in a Vessel-on-Chip model, highlighting the causative role of systemic factors and offering a platform for studying severe COVID-19 pathophysiology and therapeutic interventions.

## Graphical abstract

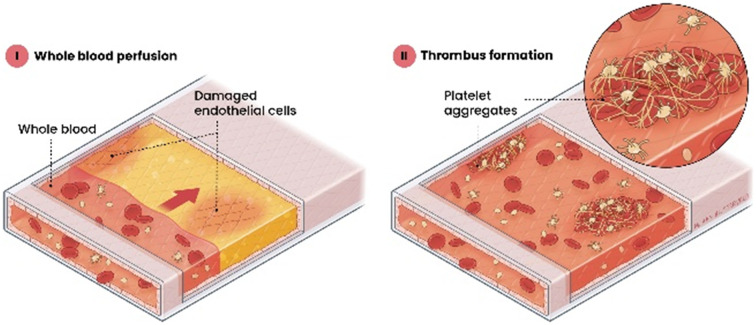

## Introduction

Since the outbreak of coronavirus disease 2019 (COVID-19), more than 750 million cases and over 7 million deaths have been reported worldwide.^1^ A subset of patients develops severe symptoms characterized by acute lung injury, endothelial inflammation and micro- or macrovascular thrombosis.^2–5^ Current pharmacological treatment of severe COVID-19 includes immunomodulation and anti-coagulation, suggesting that thromboinflammation, the interaction of thrombotic and inflammatory responses, contributes to disease progression.^6,7^ The inflammatory activation of vascular endothelium of the lung plays a central role in COVID-19 and the development of acute lung injury. This endothelial activation has been replicated in 2D and 3D cell culture models upon treatment with severe acute respiratory syndrome coronavirus-2 (SARS-CoV-2) viral particles and its spike proteins, as well as with plasma components of COVID-19 patients.^8–10^ Endothelial glycocalyx thickness, inflammatory adhesion molecule expression, barrier function and cytokine secretion have all been shown to be affected. Importantly, pro-thrombotic markers such as PAI-1 and VWF were also found to be upregulated in endothelial cells in response to stimulation with viral proteins or patient plasma.^9,11^

Vessels-on-Chips are engineered microfluidic cell culture models of human blood vessels that can capture multi-cell type, dynamic processes of vascular dysfunction beyond basic aspects of endothelial activation. Within the COVID-19 research domain, Vessels-on-Chip models have already been used to model endothelial activation and degradation after stimulation, specifically glycocalyx degradation,^12^ loss of barrier function,^13–16^ and immune cell recruitment.^14,17^ From earlier work, we know that Vessels-on-Chips can be used to study components of thromboinflammation simultaneously, including platelet activation, endothelial activation, coagulation, complement activation, and immune cell recruitment.^18–22^ Vessel-on-Chip systems have previously been employed to demonstrate COVID-19-related thromboinflammation.^23,24^ However, these studies were performed with well-defined stimuli like recombinant spike protein, live SARS-CoV-2 virus, or cell culture effluent, but not patient plasma samples. Stimulation of Vessel-on-Chip models with patient plasma, in the absence of direct viral infection or a local immune response, could yield novel insights into the mechanisms underlying COVID-19-related thromboinflammation. In particular, it would demonstrate to what extent circulating plasma components contribute to vessel dysfunction and subsequent microthrombosis in the context of severe COVID-19. Here, we established a Vessel-on-Chip model in which we demonstrate that plasma from hospitalized COVID-19 patients induces microthrombosis. We demonstrate that the model is able not only to replicate patient variability, but also to reveal underlying disease mechanisms, including immune cell–platelet interactions.

## Results

### Vessels-on-Chip with human pluripotent stem cell-derived endothelial cells exhibit microthrombosis in response to inflammation

**Fig. 1:**
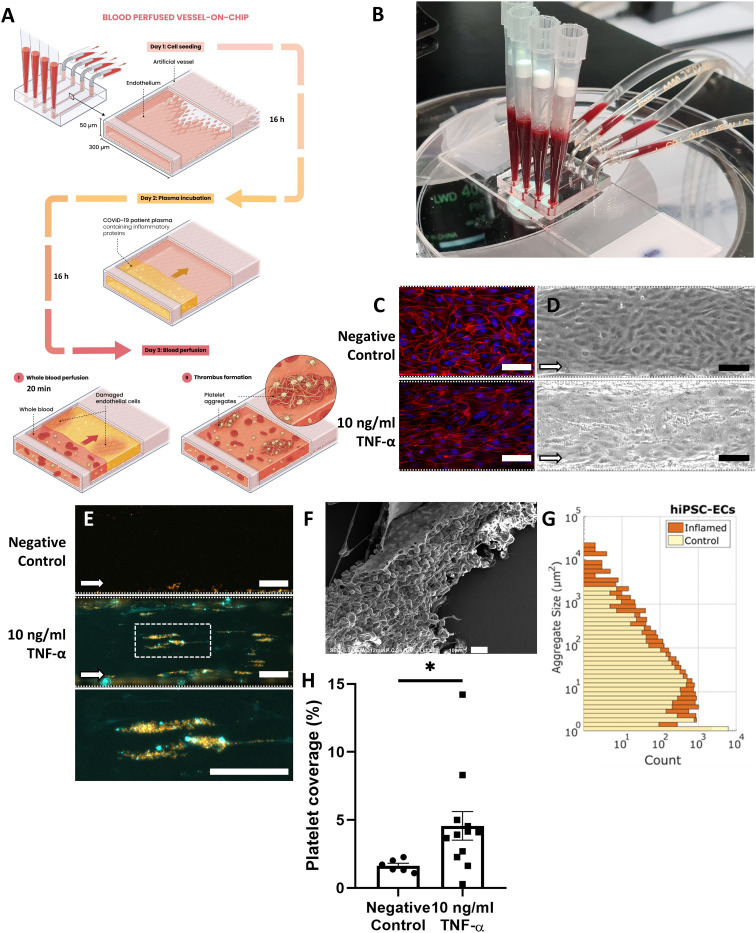
Characterization of microthrombosis in the Vessel-on-Chip upon inflammation. A: Schematic of Vessel-on-Chip exposure to cytokines or patient plasma. B: Photograph of the Vessel-on-Chip perfused with human whole blood. C: Comparison of VE-cadherin morphology of hiPSC-ECs, in control conditions and upon activation with 10 ng mL^−1^ TNF-α. Scale bar: 100 μm. D: Brightfield microscopy images of hiPSC-EC monolayers in normal and activated state after perfusion with human whole blood, leading to adhered platelets. Scale bar: 100 μm. E: Fluorescence microscopy images of adherent blood platelets (orange) and fibrin (cyan) networks in control conditions and after activation with 10 ng ml^−1^ TNF-α. Scale bar: 100 μm. F: Scanning electron micrograph of a perfused channel with whole blood shows blood clots adhering to endothelial cells. Scale bar: 10 μm. G: Platelet aggregate size measured in normal and activated conditions. H: Platelet coverage in normal (*n* = 6) and activated (*n* = 12) conditions. Aggregate size and platelet coverage are determined using image analysis of fluorescence microscopy data. Error bars show standard error of mean. * *p* < 0.05.

To recapitulate interactions between the vessel wall and blood components in thrombosis, we designed a microfluidic chip that contains multiple channels with a cross-section of 300 × 50 μm (width × height; Fig. 1A and B). The channels were lined with a confluent monolayer of human induced pluripotent stem cell-derived endothelial cells (hiPSC-ECs). These Vessels-on-Chip were then treated with the inflammatory cytokine tumor necrosis factor-alpha (TNF-α, 10 ng ml^−1^), then perfused with human whole blood (Fig. 1A and B). After 16 hours of stimulation with TNF-α, the monolayer was still intact, although F-actin was organized into stress fibres (Fig. S1†) and intercellular junctions like VE-cadherin exhibited a more jagged pattern (Fig. 1C), typical for activated endothelium.^25^

Upon perfusion with healthy human whole blood containing fluorescently labelled platelets at a physiological shear rate of 750 s^−1^, thrombi formed on the surface of the endothelium within 20 minutes (Fig. 1D). Fluorescence- and scanning electron microscopy showed that the thrombi contained both platelets and fibrin, as well as trapped red blood cells (Fig. 1E and F). The thrombi varied from small clusters of only a few platelets to larger aggregates over 20 μm in diameter (>1000 μm^2^; Fig. 1E and G), similar in diameter to microthrombi found post-mortem in lungs of COVID-19 patients.^26^ In both conditions platelets adhered near the edges of the channel, caused by edge effects of the laminar flow where the forward velocity of platelets is strongly reduced.^27^ The edges were therefore excluded from analysis. The percentage coverage of the Vessel-on-Chip surface by microthrombi, measured as the fluorescent signal from platelets, was approximately 1.5% in the negative control condition, and 5% in the condition with stimulated endothelium (Fig. 1H), both of which are in the same range as what has been reported previously for similar Vessel-on-Chip models.^20^

We used hiPSC-ECs to provide a consistent endothelial cell source for all experiments and thus here, only measure blood donor variation. In the future, this could be extended to patient-specific hiPSC-ECs, to examine variability in donor endothelium. For this reason, we carried out an extensive comparison of primary human umbilical vein endothelial cells (HUVECs), typically used in these types of studies, with hiPSC-ECs (Fig. S2†). No significant differences in platelet aggregation were found between the two cell types in both conditions, demonstrating that hiPSC-ECs can indeed be used to study microthrombus formation in Vessels-on-Chip.

### Patient-derived blood plasma induces endothelial activation that recapitulates *in vivo* vascular dysfunction

We next used the hiPSC-EC Vessel-on-Chip model to study the effects of patient plasma on microthrombosis. A group of hospitalized COVID-19 patients (*n* = 12) with diagnosis confirmed by polymerase chain reaction (PCR), was compared to unmatched healthy volunteers (*n* = 12). The sample size was determined after a power analysis based on the data shown in Fig. 1H. As a reference, we show data of two hospitalized bacterial pneumonia patients who tested PCR negative for COVID-19 (*n* = 2); this data is not included in statistical analysis due to the small sample size. The mean age of the COVID-19 patients was 62 years, 42% of the patients were male, and the average body mass index (BMI) was 30 kg m^−2^ (Table S1†). On average, COVID-19 patients were hospitalized 10 days post-symptom onset, with an average hospital stay of 7 days. Of the 12 COVID-19 patients, two required intensive care unit (ICU) admission, and one died. Venous thromboembolic complications were observed in three patients: two cases of pulmonary embolism and one case of thrombophlebitis. Bacterial pneumonia patients were hospitalized 3 and 33 days, respectively, with no thrombotic events diagnosed.

**Fig. 2:**
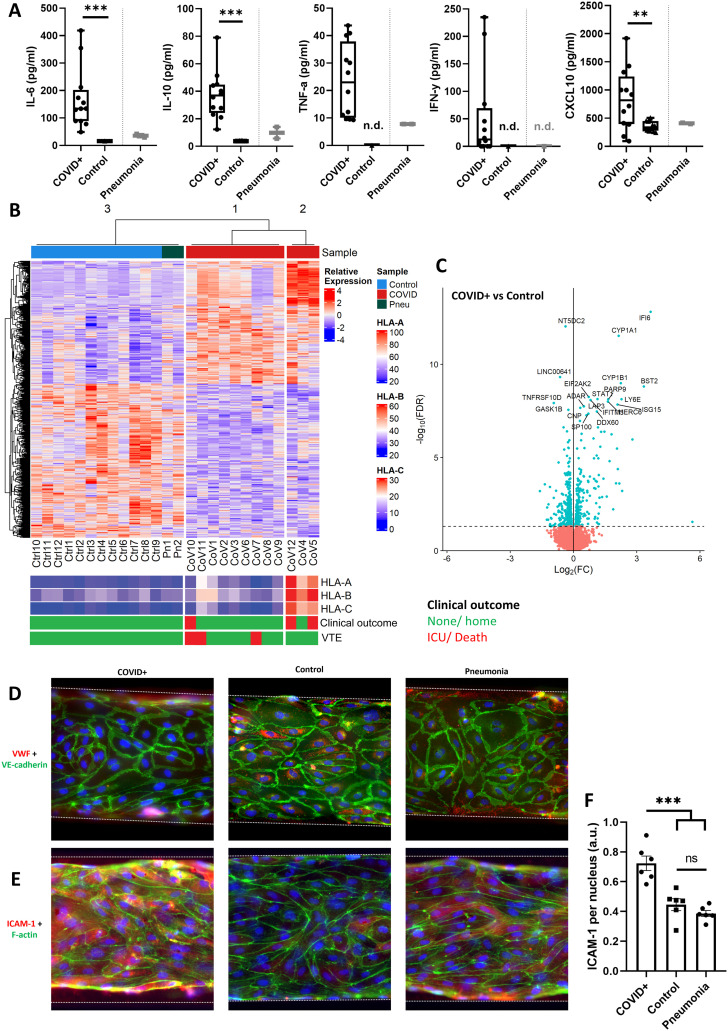
Characterization of plasma samples from COVID-19 patients and their effects on endothelial cells in the Vessels-on-Chip. A: ELISA data shows elevated plasma concentrations of the cytokines IL-6, IL-10, TNF-α, IFN-γ, and CXCL10 in COVID-19 patients, ** *p* < 0.01; *** *p* < 0.001. B: Differentially expressed gene analysis of hiPSC-ECs treated with plasma samples from healthy controls (‘Ctrl’), patients with pneumonia (‘Pn’) and patients with COVID-19 (‘CoV’). The COVID-19 samples are divided into two sub-clusters, one with low expression, and one with elevated expression of class 1 HLA genes (‘HLA-A’, ‘HLA-B’, ‘HLA-C’). Individual plasma samples are also annotated with patient clinical outcome, with severe outcomes (admission to the intensive care unit, ICU, or death) labelled in red. C: Volcano plot of differentially expressed genes comparing hiPSC-ECs treated with plasma samples from COVID-19 patients and controls. Top 20 differentially expressed genes have been marked by name (blue: differentially expressed genes; red: genes with a −log_10_(FDR) value below 1). D: Representative immunofluorescence images of VWF, VE-cadherin and DNA in Vessels-on-Chip treated with plasma from COVID-19 patients, pneumonia patients or controls. Scale bar, 100 μm. E: Representative immunofluorescence microscopy images of ICAM-1, F-actin, and DNA in all aforementioned conditions. Scale bar, 100 μm. F: Quantification of ICAM-1 shows a significant increase in expression when hiPSC-ECs are stimulated with COVID+ plasma, *** *p* < 0.001, ns = non-significant.

Plasma concentrations of the cytokines interleukin (IL)-6, IL-10, TNF-α, interferon (IFN)-γ, and chemokine (C-X-C motif) ligand (CXCL)-10 were measured using enzyme-linked immune sorbent assays (ELISA). This panel was chosen as these cytokines were known to be commonly elevated in hospitalized COVID-19 patients.^28^ Concentrations of all cytokines were significantly higher in COVID-19 patients than in healthy volunteers (Fig. 2A–E).

Vascular dysfunction was studied after stimulation of hiPSC-ECs with patient or control plasma. Recalcified plasma was added to endothelial cell culture medium (EGM-2) in various dilutions to test whether it was appropriate for endothelial stimulation in the Vessel-on-Chip model (Fig. S3†). A 1 : 4 dilution of human plasma in the medium did not induce measurable degradation of the endothelial monolayer after overnight stimulation. Higher plasma fractions have no positive effect on the cell proliferation rate when compared to the chosen dilution.^29^ Therefore, this dilution was used for all subsequent experiments. We treated hiPSC-ECs with diluted plasma overnight, isolated RNA from all 26 samples, and performed RNA-sequencing and a transcriptomics analysis. Differentially expressed gene (DEG) analysis yielded three unique clusters, with one containing all control and pneumonia samples, and two clusters containing only COVID-19 samples (Fig. 2B). The smaller COVID-19 cluster contained three samples, two of which were from patients who either died or were transferred to the ICU. This cluster exhibited an upregulation of genes associated with viral infection of the endothelial cells compared to samples in the larger COVID-19 cluster, of which class 1 human leukocyte antigen (HLA) genes were the most upregulated. As an increased class 1 HLA activity is associated with viral infection of the cells,^30^ we analysed SARS-CoV-2 RNA reads in all the samples (Table S2†). No active infection or viral replication took place in any of the treated hiPSC-EC samples, including those in the HLA-expressing cluster. Very low levels of SARS-CoV-2 RNA fragments were found in cells treated with plasma of healthy controls, pneumonia patients, and COVID-19 patients, with no noticeable differences between the clusters. This is in contrast with previous studies that found an association of SARS-CoV-2 RNA load in plasma with disease severity.^31,32^ Nevertheless, even in the absence of active infection or replication, the upregulation of genes associated with viral infection in the small cluster could potentially be caused by interaction with inactive SARS-CoV-2 particles, or even protein fragments of viral particles. Also in the clinic, increased nucleocapsid antigens in the blood stream are associated with disease severity, if sampled within 8 days after symptom onset.^33^

When comparing RNA isolated from hiPSC-ECs treated with COVID-19 plasma to those treated with plasma from healthy donors, we observed that the DEGs (Fig. 2E) were primarily linked to activation of the innate immune response in general, and interferon signalling in particular. Interferon signalling is associated with the prevention of cellular infection.^34^ This activation of the innate immune response upon treatment with plasma from COVID-19 patients is also reflected in the ontologies of the most significantly upregulated/downregulated genes (Tables S3 and S4†). Our findings are in line with multiple other studies that have analysed the effect of COVID-19 infections in various types of endothelial cells.^35–42^

In addition to notable changes in gene expression patterns associated with the innate immune system, our data also showed a change in the expression of genes associated with wound healing and platelet activation pathways (Fig. S4†). To highlight, the von Willebrand factor (VWF) is associated with platelet adhesion to endothelial cells, while upregulated CD34 is associated with the immune cell recruitment through its interaction with L-selectin. Furthermore, signal peptide-CUB-EGF domain-containing protein 1 (SCUBE1) is a gene that has previously been associated with thrombotic complications in COVID-19 patients, due to its active role in platelet aggregation.^43^ These transcriptomic changes were supported by immunofluorescence microscopy, in which Vessels-on-Chip stimulated with COVID-19 patient plasma exhibited discontinuous intercellular junctions, loss of intracellular VWF from the endothelial cells, increased expression of the cellular adhesion molecule ICAM-1, and formation of F-actin stress fibres (Fig. 2D–F). These cellular changes are hallmarks of endothelial activation in an inflammatory context.^25^

### Platelet aggregation is increased by plasma of COVID-19 patients

To study whether the observed transcriptomic and immunofluorescent changes in the hiPSC-ECs also lead to microthrombosis, we perfused Vessels-on-Chip with healthy donor whole blood after stimulating them overnight with plasma from COVID-19 patients. After stimulation, channels were examined for cell monolayer integrity, and perfused with recalcified human whole blood at a shear rate of 750 s^−1^. Blood-perfused Vessels-on-Chip were analysed by measuring the fluorescence intensity of labelled platelets. All plasma donors were tested three times, always using different whole blood donors to account for biological variance of used donor blood.

**Fig. 3:**
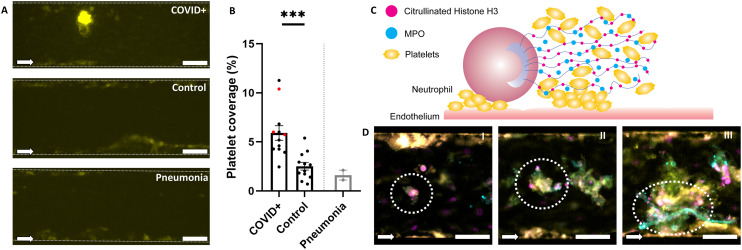
Vessels-on-Chip treated with plasma from COVID-19 patients exhibit elevated platelet aggregation. A: Representative fluorescence microscopy images of three Vessels-on-Chip after perfusion with CD41-labelled human whole blood. All conditions showed platelet adhesion to the sides of the channel (yellow), but only in the Vessel-on-Chip treated with COVID-19 plasma (‘COVID+’) were dense clots formed in the middle of the channel. Scale bar, 100 μm. B: Mean platelet coverage in Vessels-on-Chip treated with plasma from COVID-19 patients (‘COVID+’), healthy controls (‘control’), and pneumonia patients (‘pneumonia’). Patients with VTE are indicated by red datapoints. Error bars: SEM, *** *p* < 0.001. C: Schematic representation of the process of NETosis, where neutrophils interacted with activated endothelium and associated platelet aggregates to release their nuclear contents. Their DNA strands are structured with citrullinated histone H3 (H3cit), and contain antiviral proteins like myeloperoxidase (MPO). The sticky DNA creates new areas to which platelets adhere, further increasing the size of aggregates. D: Representative images of different stages of NETosis as identified in Vessels-on-Chip treated with plasma from COVID-19 patients. Formed NETs show different morphologies of proteins CitH3 (magenta) and MPO (cyan), from minor (I) to more severe (II and III) patterns of secreted DNA and MPO, eventually forming web-like structures to which CD41-positive platelets (yellow) can bind. Scale bar, 100 μm.

Blood-perfused Vessels-on-Chip stimulated by healthy donor plasma did not show elevated platelet aggregation when compared to Vessels-on-Chip incubated with culture medium (Fig. 3A and B). Incubation of Vessels-on-Chip with plasma of COVID-19 patients led to significantly higher platelet coverage in the blood perfusion assay compared to incubation with control plasma. There was no significant correlation between platelet coverage and severe clinical outcomes (*P* = 0.544; Fig. S5A†), nor was there a significant difference between samples of the two COVID-19 transcriptomic sub-clusters in terms of platelet coverage (*P* = 0.222; Fig. S5B†). Additionally, patients with venous thromboembolism (VTE) did not show a significant difference in platelet coverage compared to patients without VTE (*P* = 0.333; Fig. S5C†). These results show that our Vessel-on-Chip can distinguish between diseased and non-diseased groups susceptible to microthrombosis, but does not provide additional insights into different patient risk groups. However, the comparisons between groups of COVID-19 patients had a limited sample size in the severe (*n* = 3), HLA+ (*n* = 3), and VTE (*n* = 3) clusters.

To understand the mechanisms of increased microthrombosis in Vessels-on-Chip after treatment with plasma from COVID-19 patients, we analysed neutrophil extracellular traps (NETs). Formation of NETs (NETosis) is a specific response of neutrophils upon exposure to microbes within the blood stream. Due to endotheliitis and elevated cytokine levels, COVID-19 patients are susceptible to NET formation, and their presence has been correlated with decreased survival rates.^44,45^ When NETs are formed, they can induce platelet activation and coagulation activation, leading to further platelet aggregation in combination with the activated endothelium (Fig. 3C).^46,47^

Immunofluorescent labelling to map the NET formation markers citrullinated histone H3 (CitH3) and myeloperoxidase (MPO) in the Vessel-on-Chip after patient plasma treatment and whole blood perfusion indeed showed the formation of NETs with surrounding platelet aggregates (Fig. 3D). We could not determine if the NETs formed first, or the aggregation of platelets caused NETosis, since we only assessed statically after blood perfusion had taken place. Different forms of NETs were found after blood perfusion, reflecting the dynamic interaction of inflamed endothelium and neutrophils.^48^ The first form was a trapped neutrophil that started to become active, as shown by the bright spot of CitH3 within the neutrophil, with some aggregated platelets surrounding the neutrophil (Fig. 3D, panel I). A second form showed a more diffuse pattern of CitH3, with MPO surrounding the neutrophil, as well as more platelets (Fig. 3D, panel II). In the third form there were long strands of unravelled DNA emerging from the neutrophil, evidenced as strings positive for MPO and CitH3. These strings were surrounded with large amounts of platelet aggregates (Fig. 3D, panel III). These results demonstrated that our Vessel-on-Chip not only recapitulates microthrombosis on inflamed endothelium upon treatment with COVID-19 patient plasma but also captures the prominent role of NETosis in this process.

## Discussion

We have shown that blood-perfused Vessels-on-Chip lined with hiPSC-ECs and exposed to patient plasma recapitulate COVID-19 related microthrombosis and capture key mechanistic elements like NETosis. We found a significant elevation in platelet aggregation within the COVID-19 treated vessels compared to healthy donor plasma. To date, most platelet aggregation studies in Vessels-on-Chip have been performed with primary endothelial cells like HUVECs^20,49,50^ or patient derived blood outgrowth endothelial cells (BOECs)^51^ but we showed here by direct comparison that hiPSC-ECs show similar thromboinflammatory responses in Vessel-on-Chip and are thus a useful renewable cell source for repeated studies. Since hiPSC-ECs and blood samples could, in principle, be derived from a single donor a fully patient-specific Vessel-on-Chip model could be developed in the future. This would allow long-term blood-perfusion studies without HLA-driven leukocyte activation or single-person clinical trials.^20,52^

Our transcriptomics data showed donor group-specific changes in RNA expression patterns in endothelial cells upon exposure to plasma samples, with plasma samples from COVID-19 patients showing gene expression patterns related to interferon signalling, and innate immune response. Furthermore, our dataset shows two distinct clusters within the group of plasma donors with COVID-19, showing the heterogeneity of patients within our relatively small dataset. Extending the number of samples in this study to further investigate the transcriptomic differences between these two subsets could provide further segregation between the subpopulations. Having clearer subpopulations could then provide possible biomarkers, and further mechanistic insights into why certain patients develop more severe disease or, as importantly, why some patients have higher thrombosis risk.^53,54^

Vessel-on-Chip models have been used previously to show stimulation of endothelial cells with patient-specific plasma,^12^ and to recapitulate COVID-19 related thromboinflammation with recombinant spike protein.^23^ Others have incorporated pooled COVID-19 plasma and observed changes in endothelial activation and permeability.^13,55,56^ In line with these studies, our immunofluorescence microscopy data showed changes in protein expression typical of endothelial activation. Our data did not include protein expression data per-sample, due to limited scalability of the system but most Organs-on-Chips in fact are unable to scale to the same extent as conventional well-plate cultures.^10^ However, whilst standard cell cultures have contributed to understanding COVID-19 disease mechanisms and drug target development, Organ-on-Chip models incorporating fluid flow, as here, enable the effects of shear stress on endothelium to be captured, revealing other features of the disease. The trade-off between the systems shows how both types can be complementary, one providing rapid analysis of large datasets, and the other more in-depth analysis of mechanisms. Throughout the COVID-19 pandemic, multiple Organs-on-Chips have shown their ability to provide significant insights into disease mechanisms within different organs.^15,57–62^

Our findings reinforce the observation that COVID-19 patients develop microthrombosis and thromboinflammation, key features of COVID-19 pathophysiology. We showed that circulating plasma components alone are sufficient to induce these hallmarks of the disease *in vitro*, even in the absence of active virus or local immune response, which are known to be drivers of disease progression.^63^ Our model can isolate specific contributors to the pathophysiology of COVID-19, providing mechanistic insight to indirect toxicity caused by COVID-19.^64^ The contribution of circulating plasma factors to molecular and cellular pathophysiology had been characterized in other studies,^12,65^ and we have now demonstrated that these effects also can drive endothelial dysfunction resulting in microthrombosis.

While we found an overall difference in platelet aggregation between Vessels-on-Chip treated with plasma from COVID-19 patients and healthy control donors, we found no underlying differences between subpopulations based on transcriptomics, viral reads in plasma, or clinical outcome. It is possible that elevated platelet aggregation is typical within a subpopulation of the COVID-19 group,^66^ but due to the relatively small sample size of our COVID-19 cohort, we were unable to detect well-defined differences between subpopulations. Additionally, the healthy donors were younger than the COVID-19 patients, with limited data on their comorbidities. These differences may confound results, as non-COVID factors could contribute to endothelial activation. This limitation highlights the need for future studies with age- and comorbidity-matched non-COVID controls to confirm that the observed prothrombotic phenotype is specific to COVID-19. The model could also be used to gain mechanistic insights in other poorly understood diseases where thromboinflammation underlies disease outcome, for example as in post-COVID,^67^ sepsis,^68^ or cytokine release syndrome.^69^

NETs are known as an important contributor to thromboinflammation in COVID-19 patients.^70,71^ The mechanisms and mediators of NETosis have been reported and characterized, and Organs-on-Chips that recapitulate the mechanisms and the possible adverse events that occur due to NETosis have been reported.^72–75^ The Vessel-on-Chip model described here could eventually be used to further study how, exactly, NETosis contributes to microthrombosis in COVID-19, for example by studying rolling of neutrophils, before NETosis takes place.^76^ An assay that recapitulates the full mechanism of NETosis in COVID-19 dynamically also opens opportunities to test different drugs and their ability to target NETs specifically.

In summary, we showed that blood-perfused Vessels-on-Chips lined with hiPSC-ECs can recapitulate vascular pathophysiology related to COVID-19. We extensively characterized the use of hiPSC-ECs in the model since they provide the opportunity to eliminate batch-to-batch variability commonly arising from the use of primary cell types. We furthermore demonstrated the use of diluted patient plasma to induce thromboinflammation in hiPSC-ECs, showing a change in endothelial phenotype after diseased patient plasma stimulates the cells overnight. This inflamed phenotype resembles COVID-19 induced microthrombosis, increasing measured platelet aggregation within the Vessel-on-Chip. Our model could also capture dynamic disease mechanisms related to microthrombosis in the form of NETosis. This will be useful to explore (patho)physiologies of different diseases, taking into account patient-to-patient variability, with personalized models to assess disease outcome and treatment plans.

## Methods

### Chip fabrication

A mould for soft lithography was fabricated using standard photolithography techniques. First, a silicon wafer was patterned with an SU-8 (Microchem) layer, by spin-coating negative photo resist followed by local UV exposure using a photomask, post-baked and developed, which resulted in a mould with 51 μm high features representing the microfluidic channels. Soft lithography was performed according to standard protocols. Briefly, polydimethylsiloxane (PDMS, Sylgard 184, Dow Corning) was mixed at a 1 : 10 ratio (w/w) of curing agent to elastomer, degassed, poured on the mould and cured overnight at 65 °C. The microfluidic devices were cut to size and 1 mm diameter inlets/ outlets were punched using a biopsy punch (Robbins Instruments). Simultaneously, glass microscopy slides (Epredia) were coated with degassed PDMS, using a spin coater (Spin150, APT GmbH) at 500 rpm for 5 seconds and 1500 rpm for 30 seconds, and cured overnight at 65 °C.

The cut PDMS devices were bonded to the coated microscope slides by pressing them together after activation of the respective surfaces with air plasma treatment (CUTE, Femto Science, 50 W, 50 kHz, 40 seconds exposure). Immediately after bonding, the microfluidic channels were surface-treated with 2 mg ml^−1^ dopamine hydrochloride (Sigma) diluted in tris hydrochloride (pH 8.5, Sigma) and incubated at room temperature for 2 hours. The polydopamine was subsequently flushed using MilliQ (18.2 MΩ) and 70% ethanol, after which the chips were air dried and stored at room temperature until further use. Before cells were added to the chips, channels were coated with 0.1 mg ml^−1^ collagen-1 (rat tail, BD Biosciences) diluted in DPBS (Gibco) and incubated for 30 minutes at 37 °C. After incubation, channels were flushed with EGM-2 and cells were added.

### Cell culture

Human induced pluripotent stem cells (hiPSCs, lines LUMCi0054-A and FLB6) were provided by the Leiden University Medical Centre (LUMC). Line LUMCi0054-A was typically used during the experiments, except for the initial characterization study where FLB6 was used. These stem cell lines have been defined and registered in the human Pluripotent Stem Cell registry (https://hpscreg.eu/cell-line/LUMCi001-A, https://hpscreg.eu/cell-line/LUMCi028-A). The karyotype of the cell lines has been analyzed by G-banding, and the cell lines show spontaneous *in vitro* differentiation to endoderm, mesoderm and ectoderm.

The differentiation was done using previously reported protocols.^77^ In short, human kidney epithelial cells (LUMCi0054-A) and human skin fibroblasts (FLB6) were reprogramed to hiPSCs using a non-integrative Sendai virus and redirected to endothelial fate by growing the hiPSCs on Matrigel and exposing the hiPSCs to medium containing bone morphogenetic protein 4, activin A, CHIR99021 and vascular endothelial growth factor (VEGF) followed by culture in VEGF and transforming growth factor-β inhibitor (SB431542) supplemented medium. Using magnetic CD31-specific Dynabeads (Invitrogen) only differentiated ECs were extracted from the heterogeneous culture. The expanded ECs were frozen in 40% EGM-2 (Promocell), 10% dimethyl sulfoxide and 50% fetal calf serum (v/v) to be thawed upon use. Differentiated endothelial cells have previously been extensively characterized using FACS, immunofluorescence and RNA sequencing, and have a distinct phenotype that mostly resembles an embryonic state, but also shows signs of an arterial phenotype.^77^

HiPSC-ECs were expanded human endothelial serum-free medium (EC-SFM, Gibco) supplemented with 1% platelet poor plasma (human, Sigma), 30 ng ml^−1^ VEGF (Recombinant Human VEGF 165, Miltenyi Biotec) and 20 ng ml^−1^ bFGF (Human FGF-2, Miltenyi Biotec) in a T75 coated with 0.1% (w/v) gelatin (porcine skin type A, Sigma Aldrich) solution. A confluency of 80–90% was typically reached within 48–72 hours, after which hiPSC-ECs were disassociated using Tryple (Gibco). HiPSC-ECs were centrifuged for 3 minutes at 300 × *g* and the pellet was re-suspended in EGM-2 (PromoCell) resulting in a final concentration of 15 × 10^6^ cells per ml. The microfluidic channels were lined with hiPSC-ECs by introducing 5 μl of cell suspension followed by an incubation step of 90 minutes. During incubation, disassociating of a second batch of cells was performed to line the top of the channel, which was achieved by incubating the microfluidic chip upside-down after injection of the cell suspension. After both the top and bottom of the microchannels were lined with cells, fresh EGM-2 was added to the channels using 200 μl filtered pipette tips (Starlab TipOne), which were put on a rocking platform (12°, 1 minute interval, OrganoFlow, Mimetas) overnight.

Cells typically reached confluency in the channel after overnight incubation. When the channels reached full confluency, stimulatory factors were added in the form of 10 ng ml^−1^ TNF-α diluted in EGM-2, or recalcified human blood plasma from patients. Citrated blood plasma was recalcified at 5 : 1 (v/v) with recalcification buffer that contained 1 M HEPES (Thermo Fisher), 63.2 mM CaCl_2_ (Sigma), and 31.6 mM MgCl_2_ (Thermo Fisher) diluted in MilliQ. Recalcified plasma was then diluted 1 : 4 in EGM-2 and added to channels with hiPSC-ECs. All stimulated channels were kept on a rocking platform overnight before being used for further analyses.

### Whole blood perfusion

Citrated human whole blood was provided by the Simulation and Training centre for Technical Medicine of the TechMed Centre at the University of Twente in 9 ml citrate vacuettes (Greiner Bio One) and used within 4 hours of being drawn. Blood was collected from healthy donors *via* a voluntary donor service, where repeated testing of specific individual donors was not possible. The study did not fall in the scope of the Dutch Medical Research Involving Human Subjects Act. The study was performed in accordance with the guidelines of the Declaration of Helsinki. In agreement with these guidelines, informed consent was obtained from all volunteers. Furthermore, the blood collection procedure was approved by the Medical Ethical Committee of the Hospital Medisch Spectrum Twente. Prior to whole blood perfusion, the platelets were stained using an αIIbβ3 conformation-independent CD41-FITC antibody (4% v/v, SZ22, Beckmann Coulter, IM1756U) 10 minutes prior to whole blood perfusion.^78^ The citrated whole blood was recalcified using 10% (v/v) of recalcification buffer. Two minutes prior to the introduction of whole blood, a shear rate of 750 s^−1^ (5.62 μl min^−1^), similar to arterioles and low enough to ensure that VWF does not undergo a shear-dependent structural transition,^79^ was applied using a syringe pump (Harvard PhD 2000) to ensure an established flow. A pipette tip with recalcified citrated whole blood was attached to the inlet starting the whole blood perfusion for 20 minutes, while observed with a microscope to ensure a constant flow of blood. After, a 1 minute wash using EGM-2 and fixation using 4% paraformaldehyde (v/v, Sigma Aldrich) for 30 minutes at room temperature were performed.

### Scanning electron microscopy

Blood-perfused chips were taken apart by hand, releasing the PDMS from the microscope slide. Released PDMS slabs were dehydrated using a gradient of 60, 70, 80, 90, 96, and 2 × 100% ethanol in MilliQ (v/v), for 15 minutes per step. PDMS slabs were then transferred to a container of pure hexamethyldisilazane (HMDS, Sigma) over 30 minutes. Afterwards, PDMS slabs were air dried in a fume hood overnight. Before scanning electron microscopy, samples were gold-coated using a Cressington sputter coater. Images were taken using a JEOL JSM-IT 100 SEM at an accelerating voltage of 5 kV.

### Patient criteria

Based on the data shown in Fig. 1H, we made an estimate of the sample size per population needed to show a significant difference between the COVID-19 patient group and healthy controls. In this estimate, we made the assumption that COVID-19 patients are similar in variance to TNF-α treated conditions, and healthy controls are similar to EGM-2 treated conditions. Using a sample size calculator with a confidence level of 0.95 and a power of 0.8, the needed sample size per population to reach a significant difference is 12.

In a single-centre prospective observational cohort study, three different study populations were included: patients with confirmed SARS-CoV-2 infection aged 18 years or older who were hospitalised, patients admitted with COVID-19 like symptoms in whom the diagnosis was excluded but who had a bacterial pneumonia, and healthy controls. Patient inclusion was performed *via* a standardised protocol which was approved by the Medical Ethical Committee of the Amsterdam UMC location AMC. This approval also included collecting blood samples, if patients met pre-specified inclusion criteria. The procedure was performed in accordance with the Dutch Medical Research Involving Human Subjects Act and the guidelines of the Declaration of Helsinki. All patients provided written informed consent.

Potentially eligible patients were identified *via* a screening method when patients entered the hospital *via* the Emergency Department (ED). Patients were screened for eligibility if the reported reason for the ED visit included terms regarding suspected or confirmed SARS-CoV-2 infection or pneumonia. After receiving approval by the treating physician, patients were approached for study participation.

Patients were included if they met the following inclusion criteria: age ≥ 18 years, a suspected SARS-CoV-2 virus infection, requiring additional oxygen, CRP ≥ 50 mg l^−1^, D-dimer ≥ 0.5 mg l^−1^, and were able to provide written informed consent. Patients were excluded if their medical history included venous thromboembolism, hereditary or acquired thrombophilia, or use of anticoagulant medication such as direct oral anticoagulants or vitamin K antagonist.

### Sample collection

In total, 22.1 ml of citrated blood (0.109 mM) was collected *via* antecubital vein puncture using vacutainer tubes of each inclusion. A maximum time interval of 15 minutes was used upon collecting and processing the blood samples. In order to collect the blood plasma, centrifugation was performed using a standard protocol of 2 × 20 minutes at 1560 × *g*. Afterwards the plasma was stored in a −80 °C freezer in aliquots of 200 μl each.

Next to this, clinical data was collected at baseline including: demographics, medication use, age, sex, family history of VTE, BMI, smoking status, ECOG performance status, and number of days since start symptoms of COVID-19. In follow-up, several clinical data was also collected at seven days, and thirty days after inclusion. This data included: the occurrence of VTE, bleeding, transfer to the intensive care unit and death. For data separation, COVID-19 associated adverse effects were seen as a patient that suffered a VTE, was moved to the ICU, or died.

### Plasma cytokine concentration determination

Plasma concentrations of IL-6, IL-10, TNF-α, IFN-γ, and CXCL-10 were determined in duplicate, using sandwich enzyme-linked immunosorbent assay (ELISA, BioLegend). Assays were performed according to the manufacturer's protocol. In short, 96-well plates were coated with capture antibodies in coating buffer overnight, at 4 °C. Coated plates were washed four times with 0.05% Tween-20 (wash buffer, v/v, Sigma) diluted in PBS. Then, assay diluent A was added and incubated on a plate washer for 1 hour, at 500 rpm. Plates were washed again four times with wash buffer. 100 μl blood plasma was added to each well and diluted 1 : 1 in assay diluent A if needed (IL-6, CXCL-10). Plates with plasma were incubated on a plate washer for 2 hours, at 500 rpm. Afterwards, plates were washed four times with wash buffer.

Detection antibodies were added and incubated on a plate washer for 1 hour at 500 rpm. Plates were washed four times with wash buffer and avidin-horseradish peroxidase was added and incubated on the plate washer for 30 minutes at 500 rpm. The plates were washed five times with wash buffer, with a longer soak at the fifth wash. 3,3′,5,5′-Tetramethylbenzidine (TMB) substrate was added and incubated in the dark on a plate washer for 20 minutes at 500 rpm. 1 M sulphuric acid was added to stop the reaction, and plates were analysed at 450 nm using a microplate reader (Tecan).

### RNA sequencing

HiPSC-ECs for RNA sequencing were seeded in a 6 well plate and kept in culture until a monolayer formed. Patient plasma samples were diluted in EGM-2 and treated with recalcification buffer, following on-chip protocols. After overnight stimulation, culture medium was removed and cells were treated with 350 μl of 10 mM tris(2-carboxyethyl)phosphine hydrochloride (TCEP, Sigma). RNA was isolated and purified from the samples with the Nucleospin RNA-kit (Macherey-Nagel).

RNA-sequencing and whole-genome transcriptome data were generated by Novogene (United Kingdom) on the Illumina NovaSeq6000 platform, paired-end sequencing and read length of 150 bp with a sequencing depth of 20 M raw reads per sample.

Raw data (raw reads) of fastq format were firstly processed through in-house perl scripts. In this step, clean data (clean reads) were obtained by removing reads containing adapter, reads containing ploy-N and low quality reads from raw data. At the same time, Q20, Q30 and GC content of the clean data were calculated. All the downstream analyses were based on the clean data with high quality. Reads mapping to the reference genome and gene model annotation files were downloaded from the genome website directly. The index of the reference genome was built using Hisat2 v2.0.5 and paired-end clean 1reads were aligned to the reference genome using Hisat2 v2.0.5. We selected Hisat2 as the mapping tool as Hisat2 can generate a database of splice junctions based on the gene model annotation file and thus a better mapping result than other non-splice mapping tools.

Count tables were normalised against gene length and GC content using the R-package cqn v1.4,^80^ and genes were filtered based on their expression across all the samples according to Chen *et al.*^81^ Differentially expressed genes (DEG) were identified using a general linearised model based on the package EdgeR.^82^ Benjamini and Hochberg FDR were computed to adjust *p*-values obtained for each differentially expressed gene. Using a cutoff of 0.05 at the adjusted *p*-values, we identified all up and down-regulated genes. Heatmaps were produced using the ComplexHeatmap package (2.10.0^83^), where the gene clusters were generated using the Lance–Williams dissimilarity update formula with the complete linkage method. KEGG-pathway enrichment was performed using the enrichKEGG function from the clusterProfiler package (4.2.2^84^), with adjusted *p*-values <0.05 considered to be significant according to the Benjamini–Hochberg procedure, and gene-ontology (GO) enrichment, using the goseq package (1.46^85^) using the Wallenius method and Benjamini and Hochberg adjusted *p*-values of <0.05 were considered as significant.

### Immunostaining

Chips that were not perfused with human whole blood were fixated using 4% paraformaldehyde (v/v) and then permeabilized and blocked using a solution of 1% bovine serum albumin (BSA, w/v, Sigma) and 0.1% Triton X-100 (v/v, Sigma) diluted in PBS with calcium and magnesium ions (PBS++, Gibco) for 1 hour. Afterwards, primary antibodies were diluted in the permeabilization and blocking buffer. Used primary antibodies were anti-VE-cadherin (5 μg ml^−1^, polyclonal goat, R&D systems, AF938), anti-ICAM-1 (5 μg ml^−1^, monoclonal mouse BBIG-I1, R&D systems, BBA3) and anti-VWF (10 μg ml^−1^, polyclonal rabbit, Abcam, ab6994). All primary antibodies were incubated overnight at room temperature. After primary incubation, samples were flushed with PBS++ and put on a plate washer at 100 rpm for 1 hour. Secondary antibodies were donkey-anti-goat (10 μg ml^−1^, Alexa Fluor 546, Thermo Fisher, A-11056), donkey-anti-rabbit (10 μg ml^−1^, Alexa Fluor 647, Invitrogen, A-32795) and goat-anti-mouse (5 μg ml^−1^, Alexa Fluor 647, Invitrogen, A-21235). Secondary antibodies were combined with DNA counterstaining using 4′,6-diamidino-2-phenylindole (DAPI, 12.5 μg ml^−1^, Molecular Probes, D1306). F-Actin was stained using ActinGreen 488 Readyprobes (4 drops per ml, Thermo Fisher, R37110). The second incubation step was done for 4 hours at room temperature and samples were flushed afterwards with PBS++ and put on a plate washer at 100 rpm for 1 hour.

Immunostaining of NETs was performed on fixated samples after blood perfusion. After fixation, channels were blocked using 2% BSA in PBS++ for 1 hour. Afterwards, primary antibodies for citrullinated histone H3 (R2 + R8 + R17, 10 μg ml^−1^, polyclonal rabbit, Abcam, ab5103, lot no. GR27606-1) and myeloperoxidase (10 μg ml^−1^, polyclonal goat, R&D Systems, AF3667) were incubated overnight at room temperature. Samples were flushed with PBS++ and put on a plate washer at 100 rpm for 1 hour. Secondary antibodies donkey-anti-rabbit (10 μg ml^−1^, Alexa Fluor 647, Invitrogen, A-32795) and donkey-anti-goat (10 μg ml^−1^, Alexa Fluor 546, Invitrogen, A-11056) were incubated for 4 hours at room temperature. Afterwards, samples were flushed with PBS++ and put on a plate washer at 100 rpm for 1 hour.

### Microscopy and image analysis

Fluorescence microscopy images were made with an EVOS FL Auto 2 cell imaging system (Thermo Fisher). Images were taken at either 10 × or 20 × objectives, using CY5, GFP, RFP and DAPI filter cubes. Three microscopy images of blood-perfused Vessels-on-Chip were taken per condition, analysed blind by two researchers using ImageJ.^86^ The analysis protocol started by increasing the brightness and contrast, using the automatic function within the program. Then, images were cropped by excluding the 50 μm area directly adjacent to the walls of the channels, to exclude edge effects that could result in non-uniform shear rates, leading to platelet aggregation.^27^ The manual threshold function was used with dark background, to create a black and white image where only CD41+ pixels were black (platelet coverage). The thresholded image was analysed with the Analyse Particle(s) function, to obtain a covered area percentage of CD41+ pixels.

### Statistics

Statistical analysis was performed in Graphpad Prism 8. In blood perfusion experiments, each data point is the average of three technical replicates within one experiment. In patient plasma treated blood perfusion experiments, each data point is the average of three experiments, with a different whole blood donor for each experiment. The data is shown as the mean ± standard error. Cytokine concentrations were measured twice per patient, showing the mean of the measurements. The data is shown as a box & whiskers plot, with the box representing the 5th and 95th percentile and the whiskers showing the minimal and maximum value. Tests for statistically significant differences were performed using a two-tailed unpaired *t*-test for cytokine comparisons, and a two-tailed unpaired *t*-test with Welch's correction for blood perfusion studies.

## Data availability

The source data reported in this work is available in a public repository of 4TU.ResearchData. The data is available with the following DOI: https://doi.org/10.4121/5bb846d8-ed30-4e2e-bfe3-4fb607cbe8ed.

## Author contributions

HJW: conceptualization, methodology, investigation, data curation, formal analysis, visualization, writing – original draft, writing – review and editing. TFvH: formal analysis, investigation, methodology, visualization, writing – original draft, writing – review and editing. RWJvH: formal analysis, data curation, investigation, visualization, writing – original draft, writing – review and editing. HJA: formal analysis, investigation, methodology, writing – review and editing. RH: methodology, investigation, writing – review and editing. HHTM: methodology, writing – review and editing. MLR: resources, writing – review and editing. TEvM: conceptualization, supervision, writing – review and editing. AvdB: supervision, writing – review and editing. CLM: supervision, writing – review and editing. VVO: conceptualization, resources, supervision, writing – review and editing, funding acquisition. SM: conceptualization, supervision, writing – review and editing, funding acquisition. NvE: conceptualization, methodology, resources, supervision, writing – review and editing, funding acquisition, project administration. ADvdM: conceptualization, supervision, writing – original draft, writing – review and editing, funding acquisition, project administration.

## Conflicts of interest

Authors declare no competing interests.

## Supplementary Material

LC-025-D4LC00848K-s001
