# Supplementary material for: Blood-perfused Vessels-on-Chips stimulated with patient plasma recapitulate endothelial activation and microthrombosis in COVID-19
Source: Lab Chip. 2025 Feb 25;25(7):1787–800. doi: 10.1039/d4lc00848k (PMC11877278; doi:10.1039/d4lc00848k)
Supplement: LC-025-D4LC00848K-s001 [file LC-025-D4LC00848K-s001.pdf]

Supplementary Information (SI) for Lab on a Chip.
This journal is © The Royal Society of Chemistry 2025

Supplement COVID paper

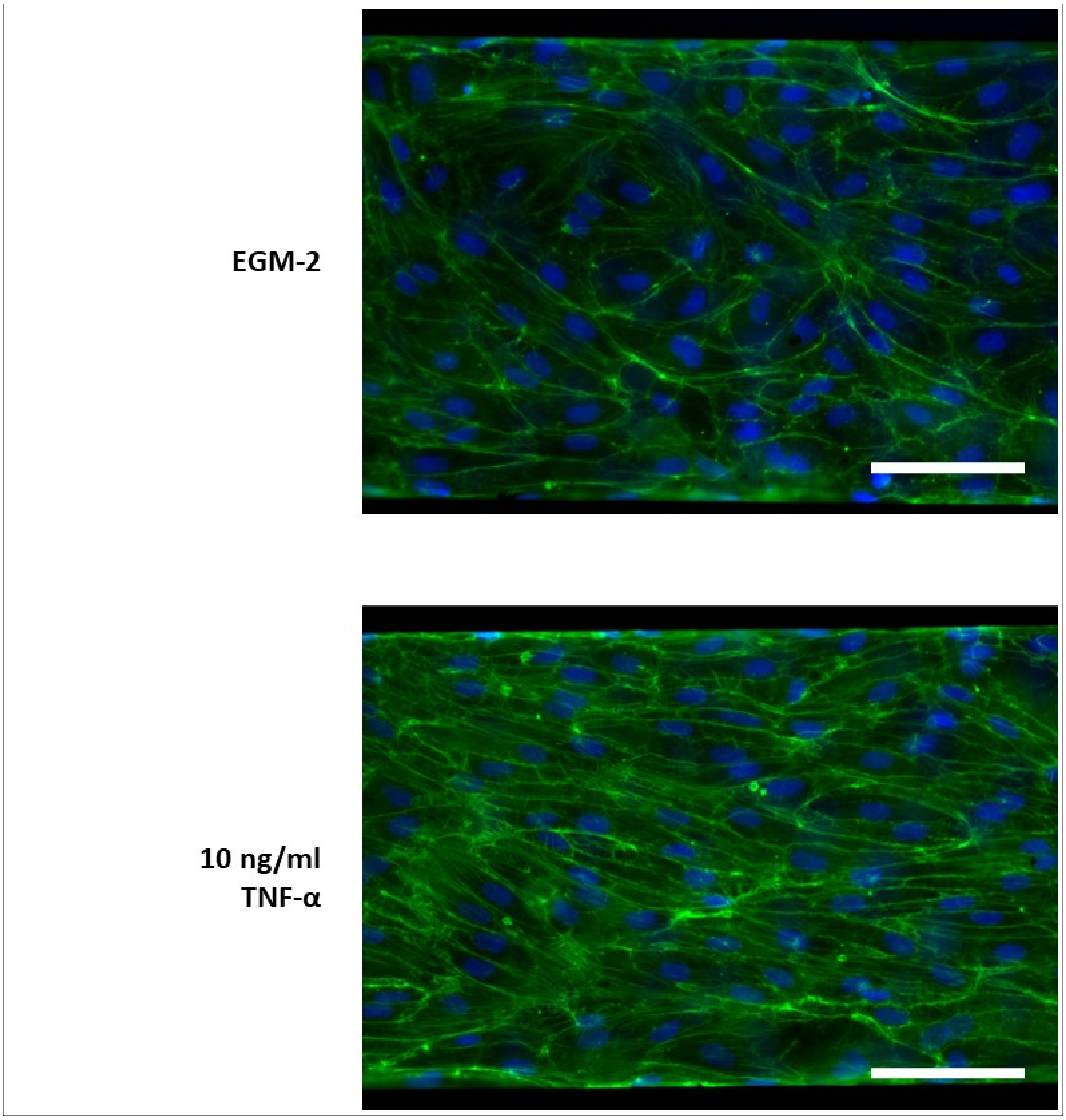

Figure S1: Comparison of Actin filaments (Green) in healthy EGM-2 vs Inflamed (10 ng/ml TNF-a) conditions. Nuclei stained in blue. Scale bar: 100 µm.

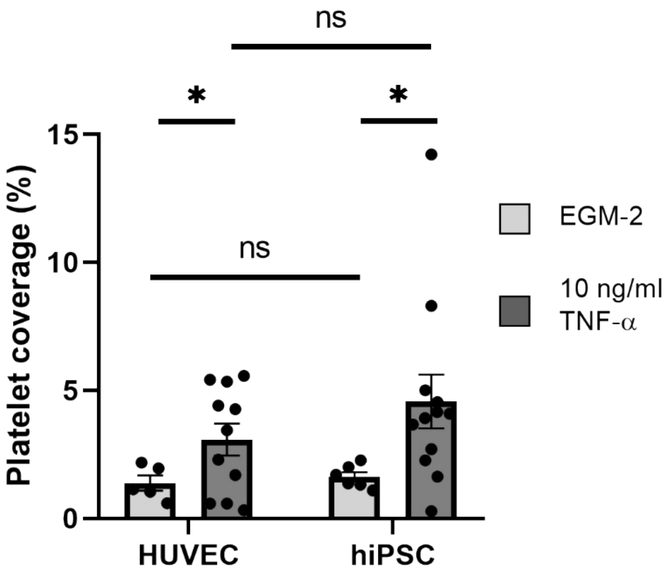

Figure S2: Platelet coverage in healthy (EGM-2) and inflamed (TNF-α) conditions in HUVECs and hiPSC-ECs. Platelet coverage is determined using image analysis of fluorescence microscopy data. Error bars: SEM. * $p<0.05$.

Table S1: Baseline characteristics of patient plasma donors

Parameter	SARS-COV-2 PCR positive (n=12)	Healthy donors (n=12)	SARS-COV-2 PCR negative (n=2)
Gender male, n (%)	5 (42)	5 (42)	0 (0)
Age, years (mean (SD))	62.50 (12.67)	25.5 (3.23)	35.50 (19.09)
BMI (mean (SD))	30.40 (7.33)	N.A.	15.93 (NA)
PCR_COVID = positive (%)	12 (100)	N.A.	0 (0)
Weight, kg (mean (SD)	91.21 (21.99)	N.A.	56.90 (12.87)
Hb level at admission, mmol/L (mean (SD))	7.92 (1.02)	N.A.	6.20 (0.14)
Lymphocytes level at admission (median [IQR])	1.11 [1.00, 1.35]	N.A.	1.51 [1.19, 1.84]
Neutrophiles at admission (mean (SD))	5.60 (2.18)	N.A.	11.39 (NA)
Platelet at admission (mean (SD))	244.00 (83.43)	N.A.	471.50 (136.47)
Creatine level at admission (mean (SD))	83.67 (31.17)	N.A.	59.50 (7.78)
D-dimer level at admission (median [IQR])	1.15 [0.60, 1.70]	N.A.	0.94 [0.94, 0.94]
CRP level at admission (mean (SD))	101.74 (64.56)	N.A.	211.10 (1.98)

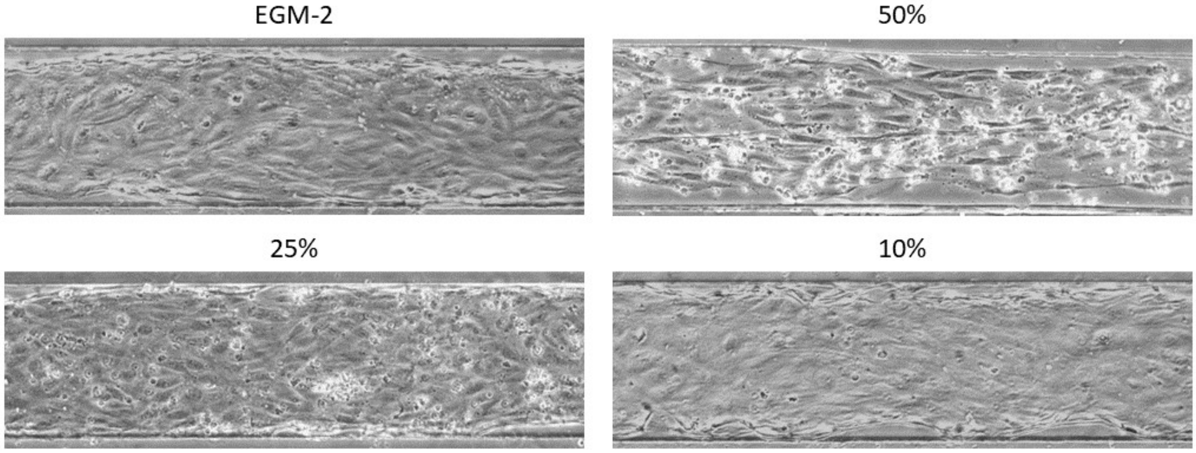

Figure S3: Monolayer comparison of different healthy plasma concentrations diluted in EGM-2 medium.

Table S2: Raw reads of homo sapiens RNA and SARS-CoV-2 RNA within stimulated HiPSC-ECs.

Sample	Raw reads homo sapiens	% reads homo sapiens	Raw reads SARS-CoV-2	% reads SARS-CoV-2
CoV 1	21914342	99,81	372	0
CoV 2	27280460	99,81	480	0
CoV 3	26288451	99,82	482	0
CoV 4	24462926	99,81	436	0
CoV 5	24583042	99,81	415	0
CoV 6	23019349	99,43	372	0
CoV 7	18660879	99,79	322	0
CoV 8	23805085	99,83	372	0
CoV 9	30513988	99,79	630	0
CoV 10	22316243	99,83	475	0
CoV 11	27827534	99,81	361	0
CoV 12	23467140	99,8	331	0
CTRL 1	26686603	99,81	382	0
CTRL 2	25813854	99,82	449	0
CTRL 3	26376322	99,78	714	0
CTRL 4	23792058	99,82	436	0
CTRL 5	25543298	99,8	375	0
CTRL 6	24486504	99,8	394	0
CTRL 7	28959628	99,8	453	0
CTRL 8	26242141	99,84	474	0
CTRL 9	27125928	99,82	420	0
CTRL 10	21293307	99,83	270	0
CTRL 11	25670817	99,8	410	0
CTRL 12	20117054	99,83	324	0
Pn 1	23168683	99,78	420	0
Pn 2	22897147	99,83	368	0

Table S3: Top 10 upregulated gene ontology terms.

	GO	Term	Ont	N	Up	Down	P.Up	P.Down
1	GO:0048525	negative regulation of viral process	BP	69	25	1	7,97E-26	0,796733
2	GO:0140546	defense response to symbiont	BP	179	35	5	1,55E-25	0,38606
3	GO:0051607	defense response to virus	BP	179	35	5	1,55E-25	0,38606
4	GO:0009615	response to virus	BP	255	39	8	3,12E-24	0,226206
5	GO:1903900	regulation of viral life cycle	BP	111	27	1	1,45E-22	0,923254
6	GO:0050792	regulation of viral process	BP	126	28	1	3,35E-22	0,945845
7	GO:0045087	innate immune response	BP	429	43	7	2,65E-19	0,861749
8	GO:0043207	response to external biotic stimulus	BP	764	57	16	2,78E-19	0,671997
9	GO:0051707	response to other organism	BP	764	57	16	2,78E-19	0,671997
10	GO:0045071	negative regulation of viral genome replication	BP	41	17	0	4,14E-19	1

Table S4: Top 10 downregulated gene ontology terms.

	GO	Term	Ont	N	Up	Down	P.Up	P.Down
1	GO:0043413	macromolecule glycosylation	BP	177	0	17	1	5,89E-07
2	GO:0006486	protein glycosylation	BP	177	0	17	1	5,89E-07
3	GO:0070085	glycosylation	BP	187	0	17	1	1,28E-06
4	GO:0005887	integral component of plasma membrane	CC	835	21	38	0,12837	3,30E-05
5	GO:0016021	integral component of membrane	CC	2881	52	95	0,731946	4,18E-05
6	GO:0031224	intrinsic component of membrane	CC	2965	55	97	0,655396	4,57E-05
7	GO:0009986	cell surface	CC	461	13	25	0,110568	5,40E-05
8	GO:0071944	cell periphery	CC	3353	68	106	0,337583	7,23E-05
9	GO:0031226	intrinsic component of plasma membrane	CC	883	23	38	0,087122	0,000109
10	GO:0023052	signaling	BP	3838	108	115	2,92E-06	0,000312

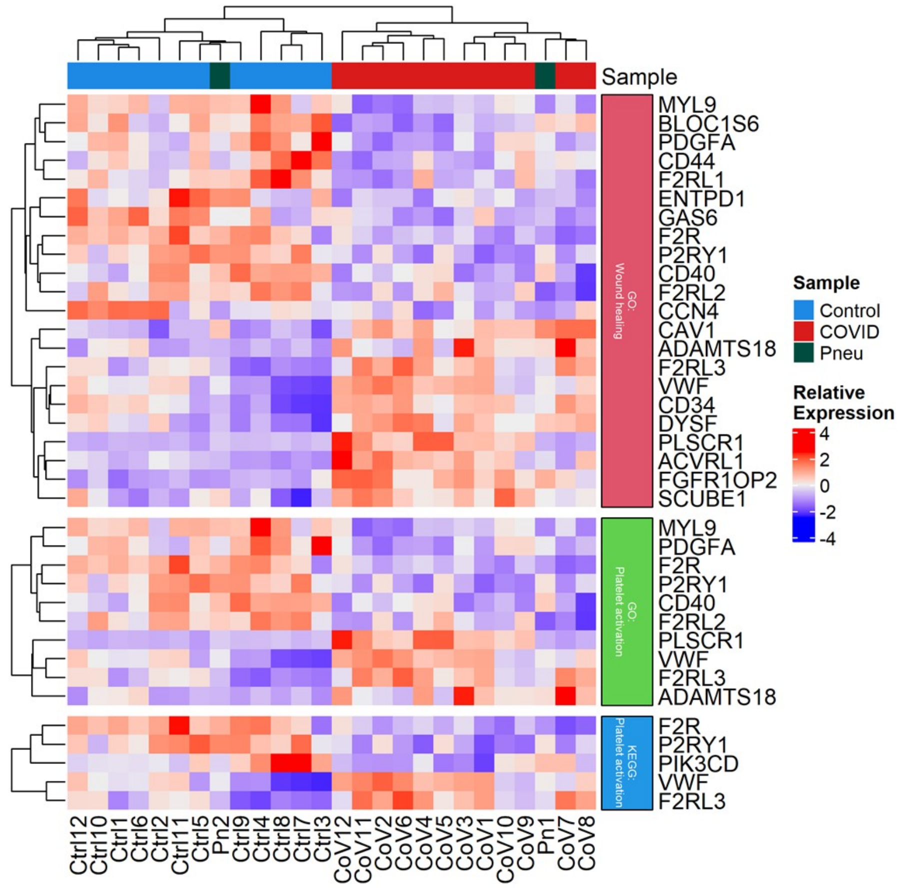

Figure S4: Gene ontologies of Wound healing and platelet activation.

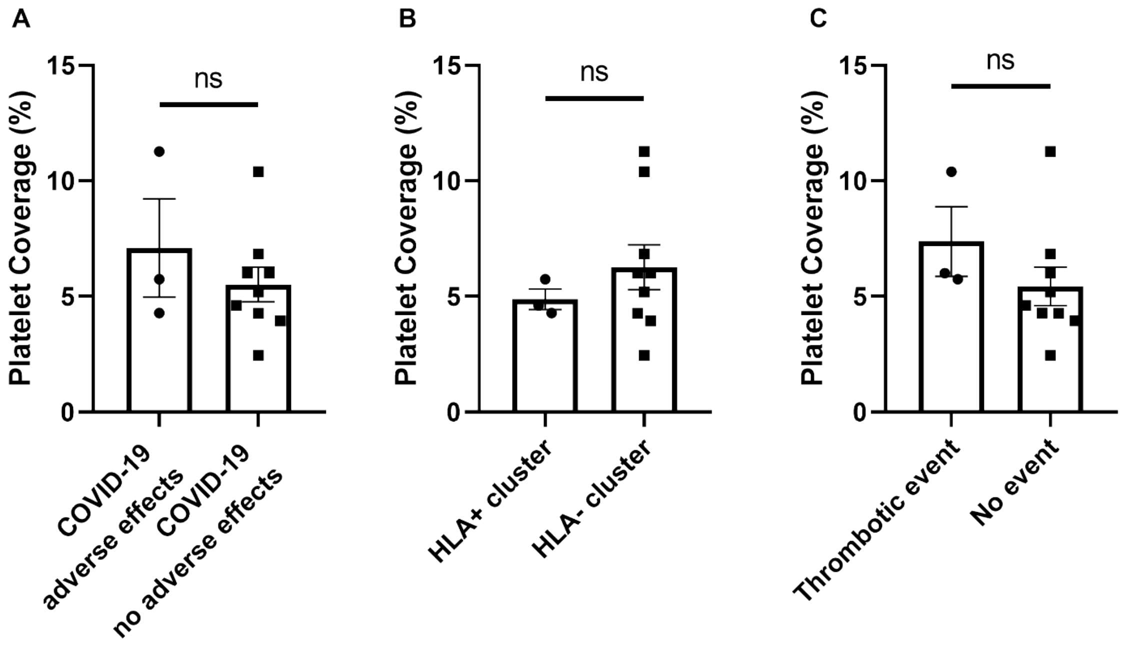

Figure S5: Platelet coverage in COVID-19 patients separated by A:adverse effects (ICU, death), B: elevated HLA class I expression, or C: Venous thromboembolism. Platelet coverage is determined using image analysis of fluorescence microscopy data. Error bars: SEM. ns: Non significant.
